# HIV dynamics linked to memory CD4+ T cell homeostasis

**DOI:** 10.1371/journal.pone.0186101

**Published:** 2017-10-19

**Authors:** John M. Murray, John Zaunders, Sean Emery, David A. Cooper, William J. Hey-Nguyen, Kersten K. Koelsch, Anthony D. Kelleher

**Affiliations:** 1 School of Mathematics and Statistics, UNSW Australia, Sydney, NSW, Australia; 2 St Vincent's Hospital, Sydney, Centre for Applied Medical Research, Darlinghurst, NSW, Australia; 3 The Kirby Institute, University of New South Wales, Sydney, NSW Australia; George Mason University, UNITED STATES

## Abstract

The dynamics of latent HIV is linked to infection and clearance of resting memory CD4+ T cells. Infection also resides within activated, non-dividing memory cells and can be impacted by antigen-driven and homeostatic proliferation despite suppressive antiretroviral therapy (ART). We investigated whether plasma viral level (pVL) and HIV DNA dynamics could be explained by HIV’s impact on memory CD4+ T cell homeostasis. Median total, 2-LTR and integrated HIV DNA levels per μL of peripheral blood, for 8 primary (PHI) and 8 chronic HIV infected (CHI) individuals enrolled on a raltegravir (RAL) based regimen, exhibited greatest changes over the 1^st^ year of ART. Dynamics slowed over the following 2 years so that total HIV DNA levels were equivalent to reported values for individuals after 10 years of ART. The mathematical model reproduced the multiphasic dynamics of pVL, and levels of total, 2-LTR and integrated HIV DNA in both PHI and CHI over 3 years of ART. Under these simulations, residual viremia originated from reactivated latently infected cells where most of these cells arose from clonal expansion within the resting phenotype. Since virion production from clonally expanded cells will not be affected by antiretroviral drugs, simulations of ART intensification had little impact on pVL. HIV DNA decay over the first year of ART followed the loss of activated memory cells (120 day half-life) while the 5.9 year half-life of total HIV DNA after this point mirrored the slower decay of resting memory cells. Simulations had difficulty reproducing the fast early HIV DNA dynamics, including 2-LTR levels peaking at week 12, and the later slow loss of total and 2-LTR HIV DNA, suggesting some ongoing infection. In summary, our modelling indicates that much of the dynamical behavior of HIV can be explained by its impact on memory CD4+ T cell homeostasis.

## Introduction

Although combination antiretroviral therapy (ART) significantly decreases morbidity and mortality it does not eradicate HIV from an individual. Despite suppressive ART over many years, HIV DNA is still found in CD4+ T cells in peripheral blood and other sites [1, 2], while HIV RNA is frequently detectable by ultra-sensitive assays [3]. Much of this residual viremia is expected to be contributed by activation of latently infected cells that were laid down over the course of untreated infection [4], but particularly established at primary infection [5]. It is also hypothesized to originate from sanctuary sites and long-lived infected cells [6, 7]. ART’s failure to clear HIV despite many years of treatment is affected by the very slowly decaying latent reservoir in resting memory CD4+ T cells [1, 8, 9], and possibly by clonal expansion of this pool through homeostatic mechanisms such as Interleukin 7 (IL-7) induced proliferation [10, 11].

Mathematical modelling has provided insights into the HIV infection processes that underlie what is observed in vivo. When linked with data on plasma viral levels (pVL), it has determined the lifespan of infected cells as well as the turnover rate of virions [12, 13]. We are now able to assay a much more diverse range of measures of HIV, such as integrated and episomal HIV DNA and cell-associated HIV RNA. The task of explaining how all these pieces of infection fit together becomes increasingly difficult, but is important if we are to achieve a fuller understanding of why HIV is not cleared with ART, and what impact might be achieved with new intervention strategies. Here we aim to produce a model that can reasonably explain the levels of pVL and HIV DNA from first infection and over many years of ART.

Others have determined models that reproduce data on pVL and cell-associated HIV over many of its phases. Funk et al. produced a model that incorporates actively, persistently, latently and defectively infected cells in order to describe pVL, total HIV DNA and gag RNA positive cells over approximately 2 years of ART [14]. Althaus et al. also incorporate actively, persistently, latently and defectively infected cells in their model simulating pVL, as well as total HIV DNA and different forms of cell-associated HIV RNA [15]. Each of these models requires different activation phenotypes of CD4+ T cells to contribute to viral levels–activated infected, persistently infected (low activation), and latently infected (resting), to reproduce the various viral phases and long-term maintenance of HIV. However these phenotypes are not directly linked to the processes that maintain activated and resting cell phenotypes for an uninfected individual, or how they are perturbed with HIV infection. This latter aspect is important as studies indicate that it is perturbed CD4+ T cell activation and proliferation that is responsible for their loss with HIV infection rather than direct killing [16]. Perturbation by HIV of the processes maintaining CD4+ T cell numbers is also likely to impact HIV dynamics especially as ART modulates antigen levels.

The mathematical model developed here to explain the different phases of HIV RNA and HIV DNA levels is based on the underlying homeostatic processes that modulate uninfected CD4+ T cell levels, and that likely contribute to HIV DNA being maintained in the different activated/resting memory CD4+ T cell subsets [17, 18]. Here we show that much of the dynamics of HIV can be explained by its impact on memory CD4+ T cell homeostasis and additionally can produce the higher infection levels observed in chronic HIV infection (CHI) compared to when ART is commenced at primary HIV infection (PHI). Under our modeling ART quickly eliminates new productive infection, with residual viremia being provided by reactivated latently infected cells which have arisen through both direct infection and clonal expansion. Since previously infected cells are not affected by antiretroviral drugs, ART intensification will have little impact on levels of residual viremia, as has been observed in a number of studies. Although ART can suppress HIV-induced increased activation and proliferation that drives much of the dynamics of infection, it does not impact homeostatic proliferation of latently infected cells which is responsible for the majority of residual viremia. Interventions capable of inhibiting clonal expansion are needed to further reduce the latent reservoir.

## Results

### HIV DNA dynamics with long-term ART

The majority of the data used in this analysis was obtained from the pilot integrase inhibitor trial (PINT) and PINT Extension studies where 8 PHI individuals (within 6 months of a negative serology or 3 bands or less on a Western blot) and 8 CHI individuals (at least 12 months of documented infection) were followed over 3 years of ART consisting of the integrase inhibitor raltegravir (RAL) and 2 reverse transcriptase inhibitors (tenofovir disoproxil fumarate and emtricitabine—Truvada). Complete descriptions of the patients and methods can be found in the PINT study reports [19–21]. In brief: all individuals were male and likely infected by HIV through homosexual intercourse; prior to enrollment they were antiretroviral naïve and their estimated duration of infection was <6 months for PHI and ranged from 1 to 23 years for CHI; levels per million CD4+ T cells of total, 2-LTR and integrated HIV DNA were obtained prior to ART and at weeks 12, 24, 52, 78, 104, 130, and 156. The episomal 2-LTR form is a non-productive form of infection that occurs at low levels but is increased following treatment with an integrase inhibitor [20, 22]. pVL was assessed with a single-copy assay.

**Fig 1:**
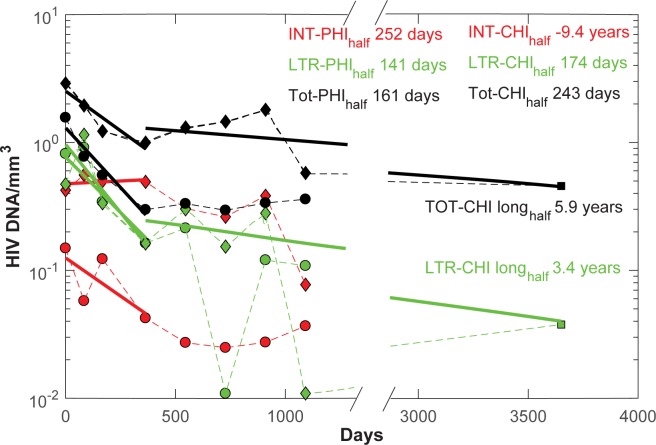
Median HIV DNA per mm^3^ changes over time, relative to commencement of ART, for PHI (circles) and CHI (diamonds). Note the break in the time axis and the regression lines between days 1000 and 3000. Regression lines for each HIV DNA component over the first year are shown (solid lines in the same color as the markers), as well as the corresponding half-lives. Assay values of total HIV DNA/ mm^3^ are shown as black markers, 2-LTR HIV DNA/ mm^3^ as green markers, and integrated HIV DNA/ mm^3^ as red markers. The two lowest 2-LTR markers are due to some of the individual values being below the assay limit of detection as well as missing CD4+ T cell counts at these later time points. Only considering the 2-LTR HIV DNA values from year 1 for CHI (but omitting the low final value) and for the year 10 time point gives a half-life of decay of 3.4 years, considerably slower than estimates of memory cell division half-life of 22 weeks but consistent with the longer division half-life of naïve cells (3.5 years) [26]. Total HIV DNA from year 1 for CHI exhibited a 5.9 year half-life. Integrated HIV DNA increased for the CHI group over the first year leading to a negative half-life.

Before we attempt to model HIV dynamics, we first describe changes in HIV DNA levels for both PHI and CHI groups with the introduction of ART. Since we will later model how HIV DNA levels are related to pVL, the latter being expressed per volume of blood, we convert HIV DNA from number of copies/10^6^ CD4+ T cells to their value per mm^3^ of peripheral blood. This also better reflects changes in HIV levels over periods where CD4+ T cell numbers increase, as will be the case under immune reconstitution with ART. As commented previously for one year of this ART regimen, 2-LTR HIV DNA levels for PHI overlapped with those from CHI, whereas both integrated and total HIV DNA levels differed significantly (Fig 1) [20, 21]. Moreover, week 12 levels of 2-LTR HIV DNA were highly elevated with this RAL-based regimen before decaying with an overall first year half-life of around 150 days. Although 2-LTR levels were quite variable, their change over time substantially slowed to the point that they were tending to levels from individuals who had been enrolled on a non-raltegravir (nonRAL) regimen for a mean of 10 years [23]. Similarly, integrated and total HIV DNA levels tended to stabilize not long after this first year to the point where total HIV DNA levels were substantially the same as for individuals after 10 years. This slowing of HIV DNA decay rates after 2 or 3 years of ART is also evident in other longitudinal studies of the latent reservoir [1, 24, 25]. The relatively unchanged levels of cells containing integrated HIV DNA over the first year for the CHI group are possibly due to a high proportion of replication defective HIV DNA, whose relatively stable dynamics differ from its replication competent counterpart, and to the slow release of these cells from lymphoid tissue as HIV levels decrease.

### Mathematical model fitted to data

**Fig 2:**
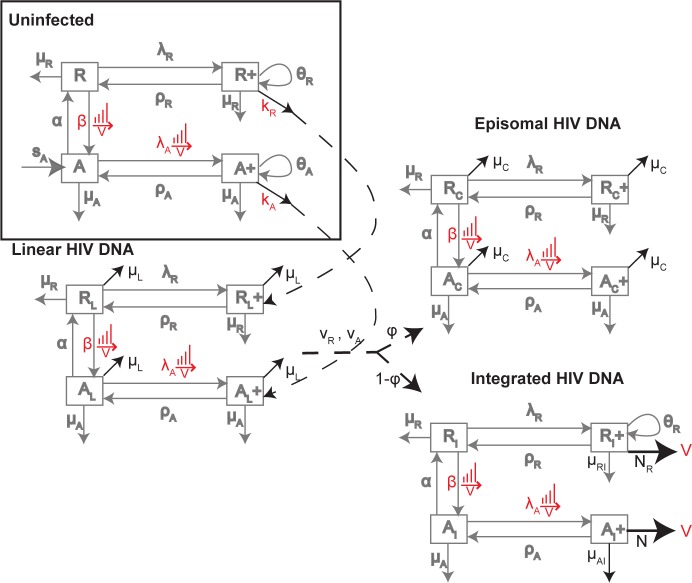
*Uninfected Homeostasis*: Mechanisms controlling resting non-dividing *R* and dividing *R*^+^, activated non-dividing *A* and dividing *A*^+^ memory CD4+ T cells numbers. Dividing cells *R*^+^, *A*^+^ arise at rates λ_R_, λ_A_ and can go through several rounds of proliferation giving rise to *θ*_*R*_ and *θ*_*A*_ copies, which can then revert to non-dividing states at rates *ρ*_*R*_, *ρ*_*A*_. Activated non-dividing cells arise from activated naïve cells at rate *s*_*A*_ and from activation of resting, non-dividing cells *R* at rate *β*. Activated non-dividing cells revert to a resting state at rate *α*. Resting and activated cells die at rates *μ*_*R*_, *μ*_*A*_. *HIV DNA infection of memory CD4+ T cells*: The model duplicates the uninfected network for each of the cells containing linear, episomal and integrated HIV DNA. Modifications to the uninfected network to describe dynamics of HIV are highlighted in black. Infection of the uninfected subsets leads initially to cells with linear HIV DNA (subscript L), then either episomal (subscript C) or integrated HIV DNA (subscript I). All parameters are the same across each of these networks. The additional parameters relate to the loss of linear and episomal HIV DNA within cells at rates *μ*_*L*_, *μ*_*C*_ respectively, progression of linear at rate *ν*_*A*_, *ν*_*R*_ in activated and resting cells respectively which convert to episomal HIV DNA with probability *ϕ* or integrated HIV DNA with probability 1- *ϕ*, a higher death rate of productively infected cells (*A*_I_^+^) at rate *μ*_*AI*_, and the rate of production of virions from these cells *N* per day. We also assume dividing resting cells with integrated HIV DNA can produce virions at rate *N*_*R*_ and die at rate *μ*_*RI*_.

To determine whether the dynamics of these cellular levels of HIV and resultant pVL could be explained by the interaction between memory CD4+ T cell homeostasis and HIV dynamics [16, 27], we generated a mathematical model that incorporated both antigen-driven and homeostatic proliferation, where the former resided within a subset of activated memory CD4+ T cells (CD38+) and the latter within a subset of resting memory CD4+ T cells (CD38-). Proliferation in response to antigen, from any background antigen or HIV once infected, will occur in the activated subset, while homeostatic proliferation occurs in response to cytokines such as IL-7 and IL-15 within the resting subset [28]. The levels of the dividing components within each of the activated and resting subsets were based on percentages of resting and activated cells expressing the proliferation marker Ki-67 [16, 17]. To be able to incorporate each of these processes responsible for CD4+ T cell maintenance for an uninfected individual, and later when HIV infection is included, we divided memory CD4+ T cells in the model into 4 subsets (Fig 2 and S1 Text): resting and non-dividing (*R*), resting and dividing (*R*^+^, encompassing cells undergoing homeostatic proliferation), activated and non-dividing (*A*), and activated and dividing (*A*^*+*^, encompassing cells undergoing antigenic proliferation). Prior to HIV infection, each of these subsets was assumed to be in steady state (S1 Table).

We overlaid the basic uninfected model of memory CD4+ T cell proliferation and homeostasis with duplicates for each of the relevant stages of HIV infection, as linear HIV DNA progresses to integration and viral production, or to the dead-end episomal stage (Fig 2). Only a fraction (*p*_*inf*_ = 0.001) of virions were considered infectious (*V*_*I*_ infectious, *V*_*NI*_ non-infectious) [29–31], and similarly a fraction of new integration events were replication defective (*p*_*def*_). We assumed HIV increased the rate of antigen-driven proliferation λ_A_ and activation from resting to activated cells at rate β, where these perturbed activation profiles drive memory cells into shorter-lived phenotypes. These processes were assumed dependent on total HIV antigen represented by *V*_*I*_ + *V*_*NI*_, in a Michaelis-Menten dependent manner; infectivity rates *k*_*R*_, *k*_*A*_ were assumed to depend similarly on levels of infectious virus *V*_*I*_.

Our previous work had identified HIV DNA at levels within activated (CD38+) memory CD4+ T cells for these patients that did not differ to those of resting (CD38-) cells, either before or after one year of ART [17]. Hence not all activated cells will be productively infected, otherwise they would have exhibited higher HIV DNA levels than their resting counterpart before ART and lower levels afterwards. Successful infection of a cell generally requires it to be proceeding through the G1 phase of the cell cycle [32]. Hence infection and virion production were assumed limited to the dividing components, of which the majority are activated (A+) but where a subset were also resting (R+) particularly within the HLA-DR+ phenotype [17]. Levels of the CD38 activation marker in CD4+ T cells have been correlated with pVL in untreated HIV infection [33]. However the various activation and reversion processes allowed all HIV DNA components to spread through the other phenotypes (Fig 2).

After accounting for the steady state cell dynamics prior to infection, plus 6 parameters fixed to literature values, there were 21 parameters to be determined by fitting to 124 data points (S2 Table). Although longitudinal data were available for most of these individuals, the model was instead fitted to the mean or median of these data for each of the PHI and CHI groups. This was done for several reasons: 1) the difficulty of tailoring the 2-LTR and integrated HIV DNA assays to each individual’s virus meant that 2-LTR data was not available for 3 PHI, and integrated HIV DNA data was not available for 1 PHI and 1 CHI while a further CHI individual did not continue in the Extension Study. Hence complete data were only available for 4 PHI and 6 CHI, with some of these values being below the assays’ limits of quantification [19]. 2) One aim was to reproduce the higher median total and integrated HIV DNA values for CHI, although these differences did not always hold across each individual [20]. 3) As well as attempting to reproduce the dynamics observed in the PINT patients, an additional aim was to have model simulations consistent with results from other studies: i) that second phase pVL was 70% lower for a RAL regimen compared to a nonRAL regimen [34], and ii) that long-term ART with a nonRAL regimen resulted in little loss of total and 2-LTR HIV DNA [23]. In total the model was simultaneously fitted to 5 different groups of patients, so that means or medians over each group were used in the fitting process.

**Fig 3:**
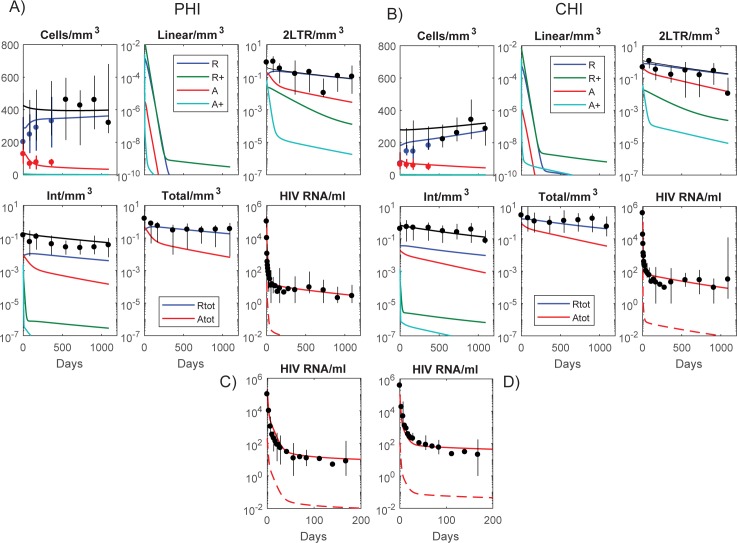
Data (means/medians over the individuals within the PHI and CHI groups, S2 Table) and model simulations for: A) and C) PHI, B) and D) CHI. Vertical lines at data points denote ranges within each group. For A) and B) total memory CD4+ T cells/mm^3^, linear HIV DNA/mm^3^, and 2-LTR HIV DNA/mm^3^ are shown as black lines while components within each cell phenotype are shown as colored lines. Cells/mm^3^ are described on a linear scale while all other panels are shown with a logarithmic y-scale. The colored lines in each of the cellular and HIV DNA panels represent values in the resting/activated, dividing/nondividing subsets as depicted by the legend in the linear HIV DNA panel. The colored lines in the integrated HIV DNA panels depict the replication competent subsets. The phenotypes are resting (*R*), resting and dividing (*R*^+^), activated (*A*), and activated and dividing (*A*^+^). In the Total HIV DNA panels each of the resting phenotypes are combined (Rtot) as well as each activated phenotype (Atot). Panels C) and D) show mean log_10_ HIV RNA copies/ml for PHI and CHI over the first 200 days—dashed lines denote the infectious components of pVL.

Fitting the model to the PHI and CHI data simultaneously, reproduced the multiphasic pVL decay seen in both groups, cellular HIV DNA dynamics, and general reconstitution of the resting memory CD4+ T cell component as activation waned with suppressed viremia Fig 3). Dynamics from first infection leading up to ART commencement are shown in S1 Fig. In general, the simulations were successful in reproducing the levels of the different HIV DNA components over the latter part of the time period but were less able to reflect the different dynamics over the first year. Although the HIV DNA simulations exhibit a slight decrease initially, as the contribution from the activated nondividing subset (*A*) wanes in comparison to that from the resting nondividing subset (*R*), this is hardly noticeable suggesting that the activated contribution may be higher than determined here, or possibly other factors such as redistribution of memory cells from lymphoid tissue is having an impact early after ART is commenced. Redistribution of cells from lymphoid tissue will also result in a larger initial increase in total memory CD4+ T cells than achieved in these simulations [35, 36]. Nevertheless this model, only consisting of memory CD4+ T cells and the homeostatic mechanisms responsible for maintaining their numbers, produces the multiphasic dynamics of pVL and the accumulation of each HIV DNA component prior to and during the administration of ART. The model can therefore provide a consistent explanation of the processes contributing to these HIV phases.

### Multiphasic pVL decay and residual viremia

The first phase drop in pVL followed the loss of productively infected cells, simulated here by the infected activated, dividing compartment (*A*_I_^+^). On the other hand, the infected resting, dividing cells (*R*_I_^+^), that are assumed to turn-over more slowly, were responsible for the second-phase pVL, thus acting as a “long-lived infected cell” component. However this still requires unintegrated linear HIV DNA to have a relatively long half-life, approximately 30 days in these simulations, significantly longer than *in vitro* and *ex vivo* estimates [37, 38]. This slow loss allowed the different point of inhibition where RAL acts in the viral life cycle to produce the 70% lower 2^nd^ phase pVL compared to a nonRAL regimen (S2 Fig) [34]. In our simulations, the 3rd phase of pVL, which constitutes residual viremia and is depicted here by the slowest decaying segment approximately after day 50, is determined by reactivation of non-dividing cells containing integrated HIV DNA, both in the resting and activated subsets. We investigate their relative contributions and the processes leading to their establishment in a later section.

### HIV DNA dynamics

Simulations suggest the loss of integrated HIV DNA during the first few years of ART follows the decay of the infected compartment within activated non-dividing cells (red lines in the Integrated HIV DNA panels of Fig 3) which decay at a rate consistent with the early loss of this component, although the simulated levels are lower. Redistribution of cells from lymphoid tissue may also play a role in these early dynamics. Activated non-dividing cells decayed with a half-life of 120 days, reflecting the 141 day half-life of integrated HIV DNA in the PHI group (Fig 1). The half-life estimates from the modelling will separate out other factors such as conversion of resting to activated cells that will contribute to the activated integrated reservoir and hence lead to an overall slower decay in this subset; on the other hand the regression estimates produce an overall half-life which is accordingly longer. The slower decay after the first few years of ART for both integrated and total HIV DNA, was determined by the rate of loss of long-lived resting, non-dividing memory CD4+ T cells with an estimated half-life of 2.3 years (Fig 1). A slower loss rate improves the fit to long-term HIV DNA values but also leads to poorer resting memory CD4+ T cell reconstitution with ART. This 2.3 year half-life leads to a greater loss of HIV DNA when compared to data after 10 years of ART, for both total and 2-LTR HIV DNA (S2 Fig, Fig 1). This is despite using a very slow intracellular clearance rate of episomal HIV DNA. Moreover the simulations do not produce the early rise in 2-LTR levels but instead assume a monotonic decay which averages out the fast rise and loss of this component of infection. It indicates that these simulations may under-represent the turnover rate of 2-LTR HIV DNA and any contribution due to redistribution from lymphoid tissue. Although homeostatic proliferation can expand cell numbers containing integrated HIV DNA, especially those that are replication defective [10, 39, 40], episomal HIV DNA will either be diluted or lost with proliferation. Previous estimates of memory T cell division occurring on average every 22 weeks [26], would produce a much faster decay of 2-LTR levels than observed here. A partial rank correlation analysis and Leave-one-out Cross-validation confirmed that simulations were very sensitive to the half-life of resting memory cells (S3 Table) and had difficulty in extracting the half-life of 2-LTR HIV DNA from the background of cell death (S4 Table). The presence of total HIV DNA and 2-LTR HIV DNA, despite 10 years of mostly non-integrase inhibitor regimens [23], and the observation that total HIV DNA predominantly reflects integrated HIV DNA levels after the first year of ART [41], suggest: i) resting memory CD4+ T cells are considerably longer-lived than a 2.3 year half-life, ii) homeostatic proliferation contributes slowly to the latent reservoir otherwise 2-LTR would be more markedly reduced in comparison to total (integrated) HIV DNA, and/or iii) there is ongoing infection.

### ART intensification has little impact on residual viremia

**Fig 4:**
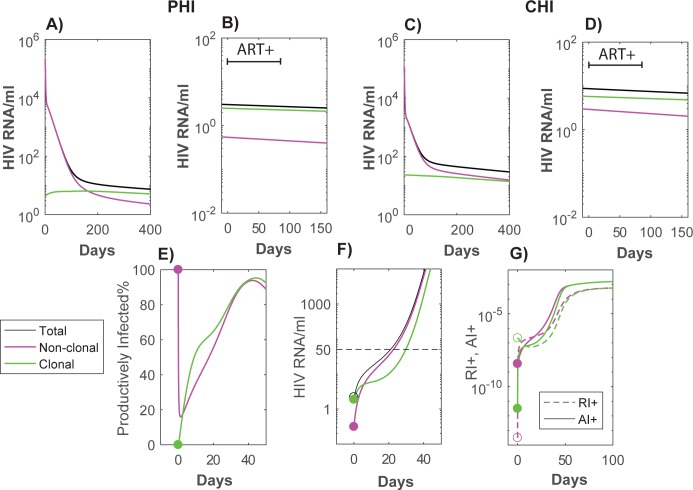
pVL simulations with ART (A and C), and treatment intensification after 3 years of ART (B and D). Panels A and B describe contributions to pVL for ART commenced at PHI, while C and D describe pVL commenced at CHI. pVL: total (black), arising from cells directly infected by virus (magenta), and from infected cells that were produced via homeostatic proliferation (through processes within the *R*^*+*^ component) from cells with integrated HIV DNA (green). In the treatment intensification panels B) and D), time has been reset to commencement of 12 weeks ART intensification (an integrase inhibitor with efficacy 99.9% is assumed added to a nonRAL regimen which had the same overall efficacy as in Fig 3 simulations), followed by reversion to the original ART regimen at day 84. E), F), G) *Rebound of pVL after stopping 6 years of ART*. Here we track the origin of the resulting viremia and cellular infection both in terms of whether the infected cell arose through clonal expansion or not, and whether the cells are in the activated dividing or resting dividing infected phenotype. E) Percentage of productively infected cells (*A*_I_^+^) of all virus producing cells (*A*_I_^+^ + *R*_I_^+^) separated into clonal (green) or direct infection (magenta) origin. Prior to interruption almost all cells producing virus are productively infected if they originally arose from direct infection, whereas if the cells arose from clonal expansion they are mostly in the resting dividing subset (*R*_I_^+^). Soon after interruption the majority of cells are productively infected regardless of their original infection type. F) Source of rebounding viremia by original infection type*–*this also tracks subsequent generations of new infection relative to these subsets. G) Expansion of virus producing cells (*A*_I_^+^, *R*_I_^+^) relative to their source of infection–as depicted in panel E) infection is mostly in the productive infected subset (*A*_I_^+^) regardless of the original source of infection.

Linear unintegrated HIV DNA decays quickly in these simulations to the point where there would be virtually no new successful infection events (Fig 3). Nevertheless, pVL is relatively constant after the first year, maintained mainly through previously infected non-dividing cells that are induced to proliferate under antigenic or homeostatic processes and become virally productive. Consistent with results from several studies [42, 43], ART intensification makes little or no impact on pVL levels, since in our simulations virus is produced from re-activated latently infected cells that already contain integrated HIV DNA. Furthermore, after sufficient time on ART, the majority of this virus is produced by cells that did not arise from a new infection event but rather through homeostatic proliferation of another latently infected cell. Simulations in Fig 4 show that the component of pVL from clonally expanded infected cells dominates after approximately 200 days from the start of ART for PHI and after 400 days for CHI, so that 12 weeks of intensification after 3 years of a standard ART regimen has no discernible impact. Under these parameter choices, after ART has sufficiently suppressed antigen-driven activation, the decay rate of viremia is determined by the life-span of resting cells (approximately with a 5.9 year half-life given the long-term dynamics of total HIV DNA). As we had found previously, these simulations produced similar proportions of HIV DNA within resting versus activated memory CD4+ T cells [17]. Note that the delay in commencement of ART for the CHI simulations resulted in the accumulation of higher levels of integrated HIV DNA (Fig 3) and of pVL (Fig 4B) and 4D)). This is due to more homeostatic generation of HIV infected cells (green lines) over the longer duration prior to ART for the CHI group, as well as a greater build-up of latently infected cells that arose from direct infection (magenta lines). For both of these reasons, earlier initiation of ART will inhibit this reservoir.

### ART interruption and viral rebound

We next investigated the interaction between the sources of residual viremia in our model simulations and rebounding virus when ART is stopped. After 6 years of nonRAL ART for the CHI group, pVL rebounded to 50 copies/ml within 21 days. Although the majority of pVL prior to interruption arose from clonally expanded cells, this was soon exceeded by viremia arising from direct infection (Fig 4F). Part of the reason for this was the segregation of the two infection types into different resting/activated compartments–the non-clonal cells producing virions were predominantly productively infected (activated and dividing) while clonal cells producing virions were almost all in a resting dividing state (Fig 4E), as might be expected from homeostasis being confined in this model to the resting compartment. This is consistent with rebounding virus reverting to an archived wild-type phenotype [44, 45]. The rebounding subset of pVL from a clonal origin would also match clonally expanded cells containing HIV DNA that were generating unspliced RNA transcripts prior to interruption [46].

## Discussion

During suppressive ART, any new infection events are outweighed by the higher number of latently infected cells becoming virally productive whether they originally arose by direct infection or from clonal expansion (Fig 4B and 4D). Such a large difference means that ART intensification will show little effect. If there is more new infection due to lower efficacy ART and higher activation levels, then some impact may be observed at least in terms of transient increases in 2-LTR HIV DNA and reduced immune activation [42]. Unless there is more ongoing infection than simulated here, RAL intensification would not significantly reduce levels of integrated HIV DNA nor its infectious component [47].

The modelling had difficulty reproducing the relatively high levels of 2-LTR and total HIV DNA levels seen after 10 years of ART [23]. These were not that much lower than levels observed after 3 years of ART in the patients from our study (Fig 1). Some of this may be attributable to the different assays used for total HIV DNA measurement. However the 2-LTR assay was the same in both studies and performed by members of our group. Model fitting to all data balanced trying to reproduce the early dynamics with these long-time total and 2-LTR HIV DNA levels. In this balance it tended to over-estimate the early data in order to not drastically under-estimate the long-term levels. Even so it required a 2.3 year half-life for resting memory CD4+ T cells, and little loss in episomal HIV DNA within cells. Additionally we could not reproduce the initial rise in these levels over the first 12 weeks of ART followed by their subsequent loss (Fig 1) as the fitting resulted in a long episomal half-life. It may be that this early rise is attributable to high levels of activated and dividing cells containing 2-LTR HIV DNA within tissue that then traffic to peripheral blood after the start of ART. Similarly the simulations of RAL intensification also failed to reproduce transient increases in 2-LTR levels at approximately 2 weeks [42] suggesting these simulations may under-estimate ongoing infection and over-estimate episomal half-life.

As with all models, be they animal, *in vitro* or *in silico*, this model contains many simplifications. It omits contributions from tissue resident cells such as macrophages [7] or T follicular helper cells [48]; it ignores contributions to pVL from sites such as the central nervous system [6]; it does not incorporate changes in cell and infection levels caused by perturbed trafficking of CD4+ T cell phenotypes with commencement of ART [35, 36]. Nevertheless it does produce a reasonable reflection of many characteristics of HIV from its onset to infection dynamics under ART, based solely on what can be observed in peripheral blood. It suggests that much of HIV dynamics observed within peripheral blood, can be explained by infection within memory CD4+ T cells and the impact HIV has on their activation and proliferation.

## Methods

### Patient data

The study protocol was approved by the Human Research Ethics Committee at St. Vincent’s Hospital (07/SVH/89). Written informed consent was obtained from each participant.

Not all cellular HIV DNA could be quantified within all patients, so for modelling purposes we only considered median HIV DNA levels for each of the PHI and CHI cohorts rather than individual data. Cellular HIV data is usually expressed as copies per 10^6^ CD4+ T cells (or PBMC) whereas pVL is stated in terms of HIV RNA copies per mL of blood. To be able to reconcile these two different units we first converted HIV DNA levels to per mm^3^ of blood, using the number of CD4+ T cells per mm^3^ for each individual, and then assuming that as 90% of HIV DNA resides within the memory phenotype [18, 49], the amount of HIV DNA within *memory* CD4+ T cells circulating through peripheral blood is given by HIV DNA/ mm^3^ = 0.9 × [(HIV DNA/10^6^ CD4+ T cells)/10^6^] × CD4+ T cells /mm^3^. The term in square brackets is the number of HIV DNA copies per single CD4+ T cell, and this is multiplied by the number of these cells per mm^3^ and attributing 90% of this to memory CD4+ T cells. Medians were calculated over the individual values for PHI and CHI.

Since the RAL regimen used in these studies produces different pVL dynamics, we also required the model to reproduce early pVL dynamics of a non-raltegravir (nonRAL) regimen [34]. Furthermore, to incorporate longer-term changes in cellular HIV DNA levels, we included median 2-LTR and total HIV DNA data from individuals after approximately 10 years on ART [23]. A full description of the ordinary differential equation model, depicted in Fig 2, is contained in S1 Text. All data were fitted simultaneously to the model simulations using a constrained optimization routine that minimized the weighted sum-of-squares error between the data and simulations for each parameter set (fmincon, Matlab 2015b, The MathWorks Inc). See S1 Text for details.

## Supporting information

S1 Text Model description and additional simulations. (PDF)

S1 Table Model parameters. (PDF)

S2 Table Data values. (PDF)

S3 Table Partial rank correlation. (PDF)

S4 Table Leave-one-out Cross-validation. (PDF)

S1 Fig Simulations for chronic HIV infection prior to ART. (PDF)

S2 Fig Simulation of a nonRAL regimen. (PDF)
