# Supplementary material for: HIV dynamics linked to memory CD4+ T cell homeostasis
Source: PLoS One. 2017 Oct 19;12(10):e0186101. doi: 10.1371/journal.pone.0186101 (PMC5648138; doi:10.1371/journal.pone.0186101)
Supplement: S1 Text — (PDF) [file pone.0186101.s001.pdf]

HIV dynamics linked to memory CD4+ T cell homeostasis

John M. Murray[1], John Zaunders[2], Sean Emery[3], David A. Cooper[3], William J Hey-Nguyen[3], Kersten K Koelsch[3], Anthony D Kelleher[3]

[1]School of Mathematics and Statistics, UNSW Australia, Sydney, NSW 2052, Australia

[2]St Vincent's Hospital, Sydney, Centre for Applied Medical Research, Darlinghurst, NSW 2010, Australia

[3]The Kirby Institute, University of New South Wales, Sydney 2052, NSW Australia

S1 Text. Model description and additional simulations

Model without HIV infection

The model of memory CD4+ T cell dynamics in the absence of HIV infection $(k_R(V) = k_A(V) = 0)$, shown diagrammatically in Fig 2, is described by the following set of 4 ordinary differential equations:

Uninfected Cells

$$\frac{dR}{dt} = \alpha A + \rho_R R^+ - (\lambda_R + \beta + \mu_R)R \tag{1}$$

$$\frac{dR^+}{dt} = \theta_R \lambda_R R - \big(\rho_R + \mu_R + k_R(V)\big)R^+ \tag{2}$$

$$\frac{dA}{dt} = s_A + \beta R + \rho_A A^+ - (\lambda_A + \alpha + \mu_A)A \tag{3}$$

$$\frac{dA^+}{dt} = \theta_A \lambda_A A - \big(\rho_A + \mu_A + k_A(V)\big)A^+ \tag{4}$$

All parameters are described in S1 Table. We assume that homeostasis results in a single division while dividing activated cells A^+ go through 6 rounds implying $\theta_R = 2$, $\theta_A = 2^6$. The model contains 9 undetermined parameters, 4 of which can be evaluated through steady state conditions of numbers of cells in each subset. We assumed there were 450 memory CD4+ T cells for an uninfected individual, 95% of which are CD38-. We had previously determined that after one year of ART, 19.5% of CD38+ memory cells expressed the proliferation marker Ki-67 while only 0.8% of CD38- cells were Ki-67+. Assuming that this level of proliferation after one year of ART roughly reflected the situation for an uninfected individual, and that the percentage of Ki-67+ CD4+ T cells is 5-fold higher than the percentage of dividing cells [1], then approximately 3.9% of CD38+ and 0.2% of CD38- memory CD4+ T cells will be dividing. We set the uninfected steady state values of R, R^+, A, A^+ to reflect these data. Hence the model of memory CD4+ T cell dynamics, equations (1)-(4) with $k_R(V) = k_A(V) = 0$, contains 5 undetermined parameters.

Mathematical Model with HIV infection

Previous investigations had highlighted the impact of perturbed activation and proliferation with HIV infection on CD4+ T cell loss [1]. Hence HIV infection was assumed to increase the rate of antigen-driven proliferation λ_A and also the activation from resting to activated β.

Not all virus, either in plasma or integrated into a cell, is infectious or replication competent. We assume virions can be either infectious V_I or non-infectious V_{NI}; as the name would suggest, we assume only infectious virus can enter cells and proceed to integration. However only a fraction of integrated virus is replication competent, capable of producing virions in either its infectious or non-infectious forms. We assume there are 2 types of cells containing integrated HIV: cells capable of producing virus denoted with a subscript I; and cells that are replication incompetent denoted with a subscript Id. Replication incompetent infected cells are assumed not to express viral proteins and so exhibit the same half-lives as uninfected cells.

We assume infection is dependent on infectious virion levels in a Michaelis-Menten manner; we also assume a similar structure for rates of activation. Hence the rates of infection of activated dividing and resting dividing cells are $k_A(V_I) = k_{A0} f_{V,inf}, k_R(V_I) = \gamma k_A(V_I)$ respectively; furthermore proliferation and activation rates are given by $\lambda_A = \lambda_{A0} + \lambda_{A1} f_V, \beta = \beta_0 + \beta_1 f_V$ where

$$f_{V,inf} = \frac{V}{1 + \frac{V}{vscale_{inf}}}$$

$$f_V = \frac{V + V_{NI}}{1 + \frac{V + V_{NI}}{vscale}}$$

This added 6 new parameters $k_{A0}, \gamma, \lambda_{A1}, \beta_1, vscale_{inf}, vscale$ to the 5 undetermined parameters from the uninfected scenario. We assumed virions were cleared at a rate of $c = 23$ per day [2]. Time was measured in days and cells and virions per mm^3 of peripheral blood for consistency. Additional to equations (1)-(4), now with $k_R(V_I), k_A(V_I) > 0$, we have the equations describing the progression of cells containing linear HIV DNA (subscript L) to either the integrated (subscript I or Id) or episomal (subscript C) forms, and the resulting virion production:

Cells with Linear HIV DNA

$$\frac{dR_L}{dt} = \alpha A_L + \rho_R {R_L}^+ - (\lambda_R + \beta + \mu_R + \nu_R + \mu_L) R_L \qquad (5)$$

$$\frac{d{R_L}^+}{dt} = (1-u) k_R R^+ + \lambda_R R_L - (\rho_R + \mu_R + \nu_R + \mu_L) {R_L}^+ \qquad (6)$$

$$\frac{dA_L}{dt} = \beta R_L + \rho_A {A_L}^+ - (\lambda_A + \alpha + \mu_A + \nu_A + \mu_L) A \qquad (7)$$

$$\frac{d{A_L}^+}{dt} = (1-u) k_A A^+ + \lambda_A A_L - (\rho_A + \mu_A + \nu_A + \mu_L) {A_L}^+ \qquad (8)$$

Cells with Episomal HIV DNA

$$\frac{dR_C}{dt} = \nu_R(\varphi + \varphi_1 u_i (1-\varphi)) R_L + \alpha A_C + \rho_R R_C^+ - (\lambda_R + \beta + \mu_R + \mu_C) R_C \qquad (9)$$

$$\frac{dR_C^+}{dt} = \nu_R(\varphi + \varphi_1 u_i (1-\varphi)) R_L^+ + \lambda_R R_C - (\rho_R + \mu_R + \mu_C) R_C^+ \qquad (10)$$

$$\frac{dA_C}{dt} = \nu_A(\varphi + \varphi_1 u_i(1-\varphi))A_L + \beta R_C + \rho_A A_C^+ - (\lambda_A + \alpha + \mu_A + \mu_C)A_C \qquad (11)$$

$$\frac{dA_C^+}{dt} = \nu_A(\varphi + \varphi_1 u_i(1-\varphi))A_L^+ + \lambda_A A_C - (\rho_A + \mu_A + \mu_C)A_C^+ \qquad (12)$$

Cells with Integrated HIV DNA

$$\frac{dR_I}{dt} = (1-p_{def})\nu_R(1-\varphi)(1-u_i)R_L + \alpha A_I + \rho_R R_I^+ - (\lambda_R + \beta + \mu_R)R_I \qquad (13)$$

$$\frac{dR_I^+}{dt} = (1-p_{def})\nu_R(1-\varphi)(1-u_i)R_L^+ + \theta_R\lambda_R R_I - (\rho_R + \mu_{RI})R_I^+ \qquad (14)$$

$$\frac{dA_\mathrm{I}}{dt} = (1-p_{def})\nu_A(1-\varphi)(1-u_i)A_L + \beta R_I + \rho_A A_I^+ - (\lambda_A + \alpha + \mu_A)A_I \qquad (15)$$

$$\frac{dA_I^+}{dt} = (1-p_{def})\nu_A(1-\varphi)(1-u_i)A_L^+ + \lambda_A A_I - (\rho_A + \mu_{AI})A_I^+ \qquad (16)$$

Cells with defective Integrated HIV DNA

$$\frac{dR_{Id}}{dt} = p_{def}\nu_R(1-\varphi)(1-u_i)R_L + \alpha A_I + \rho_R R_{Id}^+ - (\lambda_R + \beta + \mu_R)R_{Id} \qquad (17)$$

$$\frac{dR_{Id}^+}{dt} = p_{def}\nu_R(1-\varphi)(1-u_i)R_L^+ + \theta_R\lambda_R R_{Id} - (\rho_R + \mu_{RI})R_{Id}^+ \qquad (18)$$

$$\frac{dA_\mathrm{Id}}{dt} = p_{def}\nu_A(1-\varphi)(1-u_i)A_L + \beta R_{Id} + \rho_A A_{Id}^+ - (\lambda_A + \alpha + \mu_A)A_{Id} \qquad (19)$$

$$\frac{dA_{Id}^+}{dt} = p_{def}\nu_A(1-\varphi)(1-u_i)A_L^+ + \lambda_A A_{Id} - (\rho_A + \mu_{AI})A_{Id}^+ \qquad (20)$$

Virus

$$\frac{dV}{dt} = p_{inf}(NA_I^+ + N_R R_I^+) - cV \qquad (21)$$

$$\frac{dV_{NI}}{dt} = (1-p_{inf})(NA_I^+ + N_R R_I^+) - cV_{NI} \qquad (22)$$

We assumed productively infected cells were represented by activated dividing cells with replication competent integrated HIV DNA A_I^+, and these died at an elevated rate of μ_{AI} of 0.98 per day [3]. Cells containing integrated replication incompetent virus were assumed to die at the same rates as uninfected cells. A comparison of delays in effect with a non-nucleoside reverse transcriptase inhibitor compared to an integrase inhibitor had revealed a 4.6 hour time between completion of reverse transcription and integration in vivo for productively infected cells [3], so we assumed the progression rate from linear HIV DNA to either of the integrated or episomal forms mirrored this time delay with $\nu_A = 3.6$ per day. Although proliferation of cells would amplify their number it would not do so for their HIV DNA cargo unless it was integrated. We assumed cytopathic or immune effects would increase cells containing integrated HIV DNA through homeostatic division by the factor θ_R. We assumed virions were produced by productively infected CD4+ T cells, here represented by A_I^+, and by longer-lived resting dividing cells containing replication-competent virus R_I^+, but at different rates. We assumed that of linear HIV DNA that progressed to integration only $1 - p_{def}$ of these cells would be capable of producing virus. A fraction p_{inf} of virus produced by

cells containing replication competent integrated virus is assumed to be infectious with the remainder non-infectious. When the impact of antiretroviral therapy is modelled we also include the efficacy u of 2 reverse transcriptase inhibitors tenofovir and emtricitabine assumed to decrease the infectivity parameters k_A and k_R, and the efficacy u_i of the integrase inhibitor assumed to decrease the probability of integration. Integrase inhibitors increase the likelihood of blocked integration generating episomal HIV DNA [4], so we assume a fraction φ_1 of blocked integration events convert to 2-LTR HIV DNA.

The modelling simulates the different components of HIV DNA: linear unintegrated, episomal, and integrated. Integrated HIV DNA was measured directly in these patients, while one component of episomal HIV DNA was also assayed (1-LTR levels are generally not able to be directly measured). Linear HIV DNA was not assessed except as a component within total HIV DNA, but is expected to contribute little with extended time on ART [5]. Model simulations could combine all HIV DNA to obtain an estimate of total but as each of these data were assessed with a different assay their combinations do not necessarily coincide. We therefore estimated the linear combination of 2-LTR and integrated HIV DNA data that best represented total HIV DNA using linear regression over the 3 years of data of the PINT and PINT Extension studies. The optimal scaling of each HIV DNA data type to reproduce total HIV DNA (under ART) was 0.83 for 2-LTR (95%c.i. [0.01, 1.65]), and 3.0 (95%c.i. [1.66, 4.34]) for integrated, indicating perhaps that the assay under-represented integration events.

The fitting procedure was relatively insensitive to the clearance rates of unintegrated HIV DNA within a cell, since it was impacted by the clearance of the cells themselves. To limit this variability we assumed the clearance rate of linear HIV DNA molecules was at least $\mu_L \geq 1/30$. If an integrase inhibitor containing regimen exhibited efficacies of $\bar{u}$, $\bar{u_i}$ we assumed a non-integrase regimen had the same efficacy determined by $u = 1 - (1 - \bar{u})(1 - \bar{u_i}),\ u_i = 0$.

The model dynamics described by the differential equations (1)-(22) were fitted to the weighted data (S2 Table) using the parameters in S1 Table with the constrained minimization routine fmincon in Matlab 2015b, The MathWorks Inc., Natick MA, USA. At each iteration of the optimization routine, model simulations were generated using that iteration's set of parameter values, and starting with the uninfected cell levels described in S1 Table, plus an initial inoculum of infection such that there was 1 infectious virion per 10ml of blood and 100 cells in each infected compartment per litre of blood. For each optimization iteration, four model simulations were obtained using the one parameter set: 1) PHI – from infection to the commencement of ART and 3 years of the RAL regimen for the PHI group; 2) CHI – from infection to the commencement of ART and 3 years of the RAL regimen for the CHI group; 3) CHI nonRAL– from infection to the commencement of ART and 10 years of a nonRAL regimen (with the same overall efficacy as the RAL regimen); 4) CHI rebound– from infection to the commencement of ART and 6 years of a nonRAL regimen followed by one year of ART cessation. The total weighted sum-of-squares errors between the first 3 simulations and the relevant data were determined, and used as the objective value to be minimized in the optimization routine. ART cessation generally results in rapid viral rebound (HIV RNA> 50 copies/ml) [6, 7] whereas this was not always achieved by these simulations. We therefore required simulation 4 to be such that rebound occurred between days 5 and 50 after ART cessation, and incorporated this requirement as a nonlinear constraint in the constrained optimization routine.

S1 Table: Table of model parameters. Time was measured in days and cells and virions per mm^3 of peripheral blood for consistency. All rates are per day. Interquartile ranges were determined from the multiple solutions determined through the sensitivity analysis.

Data time points and weights for each of PHI and CHI

S2 Table: Table of data points used in the fitting for each of PHI and CHI. The weights used in the fitting procedure are also listed.

Untreated HIV dynamics

Infection at time 0 assumed there were 10^{-4} cells/µL in each of the infected model components (5)-(20), and 10^{-4} infectious virions/mL. Fitting only to the CHI data, but using the first time points from the PHI data, produced dynamics of untreated HIV and memory CD4+ T cells shown in S1 Fig.

S1 Fig: HIV infection occurring at day 0 and antiretroviral naïve until day 1,854 the mean estimated time for start of ART for the CHI group. Lines are simulations. The markers at the first time point (day 135) denote values prior to ART for the PHI group while the second set of markers at day 1,854 denote values prior to ART for the CHI group. Dashed lines in the HIV RNA panels denote infectious components. Coloured lines in the Integrated HIV DNA panel denote infectious components within each cell phenotype. The phenotypes are resting (R), resting and dividing (R^+), activated (A), and activated and dividing (A^+) for each of uninfected cells, or cells containing linear, 2-LTR, or integrated HIV DNA. In the Total HIV DNA panels each of the phenotypes are combined into resting (blue) or activated (red).

Simulations of a non-integrase inhibitor ART regimen

The first trials of raltegravir showed this drug suppressed pVL below a 50 copy per ml limit very quickly, mainly through a 70% lower second phase of pVL decay [8, 9]. As well as fitting the model to the PINT Extension data for CHI, we also simultaneously required a good fit to the higher second phase for pVL with a nonRAL regimen. We assumed the simulations started at the same state as for the CHI simulations using a regimen with the same overall efficacy but not including an integrase inhibitor. The higher pVL 2nd phase was achieved with a slow decay of linear HIV DNA, its progression to integrated at a 25 day half-life and the 4 day half-life of resting dividing cells containing replication competent integrated HIV DNA (S2 Fig). This progression rate, and the rate of loss of unintegrated HIV DNA, in these resting cells is much slower than the values representing a half-life of approximately 1 day determined for these molecules from in vitro experiments [10]. Although the RAL regimen suppressed pVL much more quickly, our calculations did not indicate this had a significant effect on the integrated HIV DNA reservoir, assuming equal overall efficacy.

S2 Fig: Dynamics of cells, HIV DNA and HIV RNA under a nonRAL regimen. The first 3 columns show levels over the first year while the last column shows 2-LTR and Total HIV DNA/mm^3 dynamics over 10 years of nonRAL ART – with the 10 year data points obtained from [11]. The HIV RNA data were obtained from [9], while the cell and HIV DNA data are values for the CHI group (with the RAL regimen). In the HIV RNA panel, the solid line denotes total pVL while the dashed line shows the infectious component.

Clonal expansion versus direct infection

To determine the contribution of clonal expansion to residual and rebounding viremia, the components in the mathematical model arising from clonal expansion (originally through the term $\theta_R \lambda_R R_I$ in equation (14)), were separated into their own subsets, here denoted with a z:

$$\frac{dR_{Iz}}{dt} = \alpha A_{Iz} + \rho_R R_{Iz}^{+} - (\lambda_R + \beta + \mu_R) R_{Iz}$$

$$\frac{dR_{Iz}^{+}}{dt} = \theta_R \lambda_R R_I + \theta_R \lambda_R R_{Iz} - (\rho_R + \mu_{RI}) R_{Iz}^{+}$$

$$\frac{dA_{\mathrm{Iz}}}{dt} = \beta R_{Iz} + \rho_A A_{Iz}^{+} - (\lambda_A + \alpha + \mu_A) A_{Iz}$$

$$\frac{dA_{Iz}^{+}}{dt} = \lambda_A A_{Iz} - (\rho_A + \mu_{AI}) A_{Iz}^{+}$$

$$\frac{dV_z}{dt} = p_{inf}(N A_{Iz}^{+} + N_R R_{Iz}^{+}) - c V_z$$

$$\frac{dV_{NIz}}{dt} = (1 - p_{inf})(N A_{Iz}^{+} + N_R R_{Iz}^{+}) - c V_{NIz}$$

where each of these components had value zero prior to infection. Note that no additional parameters or assumptions are used in this separation process. It merely allows calculation of the separate processes, direct infection and clonal expansion, to viremia.

Sensitivity analysis

To assess model parameter sensitivity, we generated 100 parameter sets within ±log(10) of the log (or logit) transformed optimal parameters (S1 Table), using Latin Hypercube sampling. Each of these parameter sets were then used as initial guesses and the data were refitted to generate 95 new parameter solution sets (the optimization procedure failed to converge for 5 parameter sets). A partial rank correlation of all parameters from the 95+1 solutions, determined 8 of 21 parameters that significantly impacted on the Sum-of-squares error fitting the model to the data (S3 Table). Understandably 3 of the significant parameters were related to viral infectivity ($k_{A0}, N, vscale_{inf}$). Of note is the significance of the parameter describing the death rate of uninfected (and latently infected) resting cells (μ_R). Given that the long-term total and 2-LTR HIV DNA values were relatively high, a lower death rate of these cells would lead to a better fit of these data. The parameters N and N_R essentially scale the infectious dividing component of integrated HIV DNA to virion levels in peripheral blood. Their relatively high values may be due to a combination of factors including a higher proportion of replication competent integration events than used here as well as the integrated HIV DNA assay under-reporting this component.

S3 Table: parameters and their partial rank correlation (rho) relative to model fit, listed relative to statistical significance (p). A positive Spearman's rho implies higher values of that parameter lead to higher sum-of-squares error.

We additionally assessed parameter sensitivity using a Leave-one-out Cross-validation, starting from 42 randomly generated parameter sets within ±10% of the log (or logit) transformed optimal parameter set. From each of these 42 starting parameter sets, the fitting procedure was applied with

one data point omitted (S4 Table). The omitted data points consisted of 21 values from the PHI and 21 from the CHI groups and covered: the first and last values from numbers of resting, activated and total memory cells; the first, second and last of each HIV DNA species; and 6 pVL values including the first and last values. Some data were omitted from these calculations since they were likely correlated with the included values and also to reduce computation time.

Both of these analyses highlighted the difficulty in separating the contribution of the individual components in the infectivity component with wide ranges obtained for $vscale_{inf}$. It was also difficult to extract the half-life of 2-LTR HIV DNA from this analysis against the background of cell death and transfer between activated/resting states.

S4 Table: Interquartile ranges determined using a Leave-one-out Cross-validation. All rates are per day.

References

1. Ribeiro RM, Mohri H, Ho DD, Perelson AS. In vivo dynamics of T cell activation, proliferation, and death in HIV-1 infection: Why are CD4+ but not CD8+ T cells depleted? PNAS. 2002;99(24):15572-7.
2. Ramratnam B, Bonhoeffer S, Binley J, Hurley A, Zhang L, Mittler JE, et al. Rapid production and clearance of HIV-1 and hepatitis C virus assessed by large volume plasma apheresis. Lancet. 1999;354(9192):1782-5.
3. Murray JM, Zaunders JJ, McBride KL, Xu Y, Bailey M, Suzuki K, et al. HIV DNA Subspecies Persist in both Activated and Resting Memory CD4+ T Cells during Antiretroviral Therapy. J Virol. 2014;88(6):3516-26. doi: 10.1128/jvi.03331-13.
4. Hazuda DJ, Felock P, Witmer M, Wolfe A, Stillmock K, Grobler JA, et al. Inhibitors of strand transfer that prevent integration and inhibit HIV-1 replication in cells. Science. 2000;287(5453):646-50.
5. Koelsch KK, Liu L, Haubrich R, May S, Havlir D, Gunthard HF, et al. Dynamics of Total, Linear Nonintegrated, and Integrated HIV-1 DNA In Vivo and In Vitro. J Infect Dis. 2008;197(3):411-9. PubMed PMID: 18248304.
6. Davey RT, Jr., Bhat N, Yoder C, Chun TW, Metcalf JA, Dewar R, et al. HIV-1 and T cell dynamics after interruption of highly active antiretroviral therapy (HAART) in patients with a history of sustained viral suppression. Proc Natl Acad Sci U S A. 1999;96(26):15109-14.
7. Chun T-W, Davey RT, Engel D, Lane HC, Fauci AS. AIDS: Re-emergence of HIV after stopping therapy. Nature. 1999;401(6756):874-5.
8. Markowitz M, Nguyen BY, Gotuzzo E, Mendo F, Ratanasuwan W, Kovacs C, et al. Rapid and durable antiretroviral effect of the HIV-1 Integrase inhibitor raltegravir as part of combination therapy in treatment-naive patients with HIV-1 infection: results of a 48-week controlled study. J Acquir Immune Defic Syndr. 2007;46(2):125-33. Epub 2007/08/28. doi: 10.1097/QAI.0b013e318157131c PubMed PMID: 17721395.
9. Murray JM, Emery S, Kelleher AD, Law M, Chen J, Hazuda DJ, et al. Antiretroviral therapy with the integrase inhibitor raltegravir alters decay kinetics of HIV, significantly reducing the second phase. AIDS. 2007;21(17):2315-21. Epub 2007/12/20. doi: 10.1097/QAD.0b013e3282f12377. PubMed PMID: 18090280.
10. Pierson TC, Zhou Y, Kieffer TL, Ruff CT, Buck C, Siliciano RF. Molecular characterization of preintegration latency in human immunodeficiency virus type 1 infection. Journal of Virology. 2002;76(17):8518-31.
11. Søgaard OS, Graversen ME, Leth S, Olesen R, Brinkmann CR, Nissen SK, et al. The Depsipeptide Romidepsin Reverses HIV-1 Latency *In Vivo*. PLoS Pathog. 2015;11(9):e1005142. doi: 10.1371/journal.ppat.1005142.
