# Supplementary material for: HIV dynamics linked to memory CD4+ T cell homeostasis
Source: PLoS One. 2017 Oct 19;12(10):e0186101. doi: 10.1371/journal.pone.0186101 (PMC5648138; doi:10.1371/journal.pone.0186101)
Supplement: S1 Table — (PDF) [file pone.0186101.s002.pdf]

S1 Table. Model parameters.

Time was measured in days and cells and virions per mm^3 of peripheral blood for consistency. All rates are per day. Interquartile ranges were determined from the multiple solutions determined through the sensitivity analysis.

	Parameter	Value (and interquartile ranges for the fitted parameters)	Description
Fixed	μ_{AI}	0.98	Death rate of productively infected cells (A_I^+) [1]
	ν_A	3.6	Progression rate from linear HIV DNA to integrated or 2-LTR in activated cells [1]
	c	23	Clearance rate of virions [2]
	θ_R	2	Number of cells produced through homeostasis from a single cell
	θ_A	2^6	Number of cells produced through antigen-driven proliferation from a single cell [3]
	ltr_{scale}	0.83	Multiplier of one 2-LTR HIV DNA copy contributing to total HIV DNA assay. Calculated from linear regression fitting to PINT Extension data.
	int_{scale}	3	Multiplier of one integrated HIV DNA copy contributing to total HIV DNA assay. Calculated from linear regression fitting to PINT Extension data.
	M_0	450 cells/mm^3	Number of memory CD4+ T cells per mm^3 of peripheral blood in an uninfected individual [4]
	CD38-% of M_0	95%	Mean CD38-% of CD45RO+CD4+T cells from 33 staff controls.
	Dividing percentage	0.2%	Estimated to be approximately equal to the mean percentage of dividing

	of CD38- for healthy individual		CD38- memory CD4+ T cells for the 16 individuals after 1 year of ART. Dividing cells are approximately 5-fold lower than Ki-67% [5].
	Dividing percentage of CD38+ for healthy individual	3.9%	Estimated to be approximately equal to the mean percentage of dividing CD38+ memory CD4+ T cells for the 16 individuals after 1 year of ART. Dividing cells are approximately 5-fold lower than Ki-67% [5].
	p_{inf}	0.001	Fraction of infectious virus produced by cells containing infectious integrated virus A_I^+, R_I^+. In general this is considered to range between 1/6000 and 1/100 [6, 7].
	p_{def}	0.5	Proportion of integration events that lead to defective integrated HIV DNA. A value of 0.01 was also tested but produced poorer fits.
	lin_{scale}	1	Multiplier of one linear HIV DNA copy contributing to total HIV DNA assay
Determined from uninfected steady state	λ_R	$\frac{(\rho_R + \mu_R)}{\theta_R} \frac{R_0^+}{R_0} = 6.0 \times 10^{-6}$	Rate of progression to dividing resting cells
	λ_{A0}	$\frac{(\rho_A + \mu_A)}{\theta_A} \frac{A_0^+}{A_0} = 4.6 \times 10^{-5}$	Baseline rate of progression to dividing activated cells
	α	$\frac{[(\lambda_R + \beta_0 + \mu_R)R_0 - \rho_R R_0^+]}{A_0} = 0.018$	Reversion rate of activated to resting cells
	s_A	$(\lambda_{A0} + \alpha + \mu_A)A_0 - \beta_0 R_0 - \rho_A A_0^+ = 0.42$	Generation rate of new activated non-dividing memory cells (/day/mm^3)

Fitted	k_{A0}	0.082 (0.032-0.75)	Rate of infection in activated dividing cells through Michaelis-Menten dynamics dependent on infectious pVL
	γ	0.998 (0.942-0.998)	Relative infectivity of resting dividing cells compared to activated cells
	μ_L	0.033 (0.056-0.20)	Clearance rate of linear HIV DNA molecules within a cell, $\mu_L \geq 1/30$
	μ_C	1.5×10^{-4} (1.7×10^{-5}-2.6×10^{-4}	Clearance rate of episomal HIV DNA molecules within a cell
	φ	0.50 (0.13-0.65)	Proportion of linear HIV DNA proceeding to episomal in the absence of an integrase inhibitor
	φ_1	0.85 (0.64-0.97)	Proportion of linear HIV DNA that would have proceeded to an integrated state that become episomal relative to the efficacy of the integrase inhibitor
	β_0	9.0×10^{-5} (1.6×10^{-5}-4.8×10^{-4})	Baseline activation rate of resting cells
	β_1	0.51 (6.2×10^{-4}-0.63)	Additional activation rate of resting cells
	λ_{A1}	2.0×10^{-9} (2.0×10^{-9}-3.0×10^{-8})	Additional rate of progression to dividing activated cells
	μ_R	8.3×10^{-4} (4.4×10^{-4}-2.1×10^{-3})	Death rate of resting memory CD4+ T cells
	μ_A	5.8×10^{-3} (3.3×10^{-3}-2.8×10^{-2})	Death rate of activated memory CD4+ T cells
	μ_{RI}	0.17 (0.0061-0.13)	Death rate of infected resting, dividing memory CD4+ T cells
	ρ_R	0.0052 (0.0019-0.021)	Progression rate from dividing to non-dividing resting cells

	ρ_A	0.070 (0.014-0.14)	Progression rate from dividing to non-dividing activated cells
	ν_R	0.028 (0.011-0.30)	Progression rate of linear to episomal or integrated HIV DNA in resting cells
	N	971,176 (109,481-813,224)	Rate of virion production by an infected activated dividing cell
	N_R	99,872 (10,559-70,964)	Rate of virion production by an infected resting dividing cell
	u	0.9998 (0.9986-0.9998)	Efficacy of non-RAL drugs in a RAL regimen
	u_i	0.996 (0.991-0.9995)	Efficacy of RAL
	$vscale$	0.016 (0.0037-0.075)	Half-saturation level for antigen-induced activation (virions per μL)
	$vscale_{inf}$	0.033 (0.0018-0.818)	Half-saturation level for infection (virions per μL)

References

1. Murray JM, Kelleher AD, Cooper DA. Timing of the Components of the HIV Life Cycle in Productively Infected CD4+ T Cells in a Population of HIV-Infected Individuals. J Virol. 2011;85(20):10798-805. doi: 10.1128/jvi.05095-11.
2. Ramratnam B, Bonhoeffer S, Binley J, Hurley A, Zhang L, Mittler JE, et al. Rapid production and clearance of HIV-1 and hepatitis C virus assessed by large volume plasma apheresis. Lancet. 1999;354(9192):1782-5.
3. Ahmed R, Gray D. Immunological memory and protective immunity: understanding their relation. Science. 1996;272(5258):54-60.
4. Zaunders JJ, Geczy AF, Dyer WB, McIntyre LB, Cooley MA, Ashton LJ, et al. Effect of long-term infection with nef-defective attenuated HIV type 1 on CD4+ and CD8+ T lymphocytes: increased CD45RO+CD4+ T lymphocytes and limited activation of CD8+ T lymphocytes. AIDS Res Hum Retroviruses. 1999;15(17):1519-27. Epub 1999/12/02. doi: 10.1089/088922299309801. PubMed PMID: 10580402.
5. Ribeiro RM, Mohri H, Ho DD, Perelson AS. In vivo dynamics of T cell activation, proliferation, and death in HIV-1 infection: Why are CD4+ but not CD8+ T cells depleted? PNAS. 2002;99(24):15572-7.
6. Bourinbaiar AS. The ratio of defective HIV-1 particles to replication-competent infectious virions. Acta Virol. 1994;38(1):59-61. Epub 1994/02/01. PubMed PMID: 7520666.
7. Rusert P, Fischer M, Joos B, Leemann C, Kuster H, Flepp M, et al. Quantification of infectious HIV-1 plasma viral load using a boosted in vitro infection protocol. Virology. 2004;326(1):113-29. doi: http://dx.doi.org/10.1016/j.virol.2004.05.022.
