# Supplementary material for: HIV dynamics linked to memory CD4+ T cell homeostasis
Source: PLoS One. 2017 Oct 19;12(10):e0186101. doi: 10.1371/journal.pone.0186101 (PMC5648138; doi:10.1371/journal.pone.0186101)
Supplement: S2 Table — (PDF) [file pone.0186101.s003.pdf]

S2 Table. Data values

Table of data points used in the fitting for each of PHI and CHI. The weights used in the fitting procedure are also listed.

Data	Times (Weeks relative to start of ART)	Weight*	Source
Log_{10} HIV RNA/ml	0, 0.5, 1, 1.5, 2, 2.5, 3, 3.5, 4, 6, 8, 10, 12, 16, 20, 24, 32, 40, 52, 78, 104, 130, 156	4	Means of PHI, CHI [1, 2]
Log_{10} Total HIV DNA/mm^3	0, 12, 24, 52, 78, 104, 130, 156	1	Medians of PHI, CHI [1, 2]
Log_{10} 2-LTR HIV DNA/mm^3	0, 12, 24, 52, 78, 104, 130, 156	1	Medians of PHI, CHI [1, 2]
Log_{10} Integrated HIV DNA/mm^3	0, 12, 24, 52, 78, 104, 130, 156	1	Medians of PHI, CHI [1, 2]
Log_{10} CD38+ memory CD4+ T cells/mm^3	0, 12, 24, 52	10	Means of PHI, CHI [1, 2]
Log_{10} CD38+ memory CD4+ T cells/mm^3	0, 12, 24, 52	10	Means of PHI, CHI [1, 2]
Log_{10} memory CD4+ T cells/mm^3	78, 104, 130, 156	10	Means of PHI, CHI [2]
nonRAL			
Log_{10} Total HIV DNA/mm^3	520	3	Medians [3]
Log_{10} 2-LTR HIV DNA/mm^3	520	3	Medians [3]
Log_{10} HIV RNA/ml	0, 14, 28, 56	8	Medians [4]

* Weights were chosen higher for pVL to ensure their early multiphasic dynamics were reproduced. Additionally since the model was structured on homeostasis of memory CD4+ T cells and their changes on a log_{10} scale were smaller than changes for infection values, their weights were also chosen higher.

References

1. Murray JM, McBride K, Boesecke C, Bailey M, Amin J, Suzuki K, et al. Integrated HIV DNA accumulates prior to treatment while episomal HIV DNA records ongoing transmission afterwards. AIDS. 2012;26(5):543-50. Epub 2012/03/14. doi: 10.1097/QAD.0b013e328350fb3c [doi] 00002030-201203130-00003 [pii]. PubMed PMID: 22410637.

2. Hey-Cunningham WJ, Murray JM, Natarajan V, Amin J, Moore CL, Emery S, et al. Early antiretroviral therapy with raltegravir generates sustained reductions in HIV reservoirs but not lower T-cell activation levels. AIDS. 2015;29(8):911-9. doi: 10.1097/qad.0000000000000625. PubMed PMID: 00002030-201505150-00006.

3. Søgaard OS, Graversen ME, Leth S, Olesen R, Brinkmann CR, Nissen SK, et al. The Depsipeptide Romidepsin Reverses HIV-1 Latency *In Vivo*. PLoS Pathog. 2015;11(9):e1005142. doi: 10.1371/journal.ppat.1005142.

4. Murray JM, Emery S, Kelleher AD, Law M, Chen J, Hazuda DJ, et al. Antiretroviral therapy with the integrase inhibitor raltegravir alters decay kinetics of HIV, significantly reducing the second phase. AIDS. 2007;21(17):2315-21. Epub 2007/12/20. doi: 10.1097/QAD.0b013e3282f12377. PubMed PMID: 18090280.
