# Supplementary material for: HIV dynamics linked to memory CD4+ T cell homeostasis
Source: PLoS One. 2017 Oct 19;12(10):e0186101. doi: 10.1371/journal.pone.0186101 (PMC5648138; doi:10.1371/journal.pone.0186101)
Supplement: S3 Table — (PDF) [file pone.0186101.s004.pdf]

S3 Table. Partial rank correlation.

Parameters and their partial rank correlation (rho) relative to model fit, listed relative to statistical significance (p). A positive Spearman's rho implies higher values of that parameter lead to higher sum-of-squares error.

parameter	μ_R	k_{A0}	N	λ_{A1}	$vscale_{inf}$	μ_A	β_0	u
p	5.7×10^{-8}	2.0×10^{-7}	8.4×10^{-6}	0.0080	0.012	0.024	0.028	0.031
rho	0.58	-0.56	-0.50	-0.30	-0.28	0.26	0.25	0.25
parameter	μ_C	u_i	φ_1	μ_{RI}	$vscale$	β_1	ν	ρ_R
p	0.07	0.11	0.12	0.19	0.24	0.24	0.30	0.57
rho	0.21	-0.19	0.18	-0.15	-0.14	-0.14	0.12	0.07
parameter	φ	ρ_A	N_R	μ_L	γ			
p	0.58	0.66	0.74	0.75	0.92			
rho	0.06	-0.05	-0.04	0.04	0.01			
