# Supplementary material for: HIV dynamics linked to memory CD4+ T cell homeostasis
Source: PLoS One. 2017 Oct 19;12(10):e0186101. doi: 10.1371/journal.pone.0186101 (PMC5648138; doi:10.1371/journal.pone.0186101)
Supplement: S4 Table — (PDF) [file pone.0186101.s005.pdf]

S4 Table. Leave-one-out Cross-validation.

Interquartile ranges determined using a Leave-one-out Cross-validation. All rates are per day.

k_{A0}	γ	μ_L	μ_C	φ	φ_1	β_0
0.059-0.088	0.997-0.999	0.033-0.052	2.6×10^{-5} - 1.6×10^{-4}	0.29-0.45	0.83-0.84	8.4×10^{-5} – 2.1×10^{-4}
β_1	λ_{A1}	μ_R	μ_A	μ_{RI}	ρ_R	ρ_A
0.53-0.76	2.0×10^{-9} – 9.2×10^{-9}	7.8×10^{-4} – 9.6×10^{-4}	4.3×10^{-3} – 5.3×10^{-3}	0.18-0.20	5.6×10^{-3} - 1.0×10^{-2}	0.017-0.042
ν_R	N	N_R	u	u_i	$vscale$	$vscale_{inf}$
0.017-0.027	894,052 – 988,996	98,338 – 99,139	0.9994 – 0.9998	0.995 – 0.998	0.010-0.017	0.062-0.13
