# Supplementary material for: HIV dynamics linked to memory CD4+ T cell homeostasis
Source: PLoS One. 2017 Oct 19;12(10):e0186101. doi: 10.1371/journal.pone.0186101 (PMC5648138; doi:10.1371/journal.pone.0186101)
Supplement: S1 Fig — (PDF) [file pone.0186101.s006.pdf]

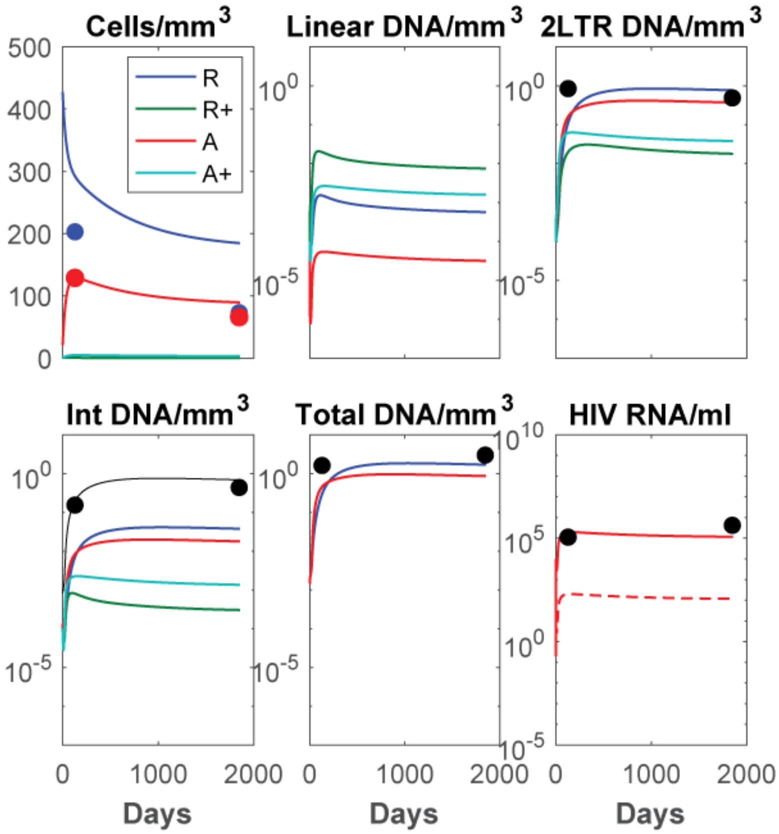

S1 Fig. Simulations for chronic HIV infection prior to ART

HIV infection occurring at day 0 and antiretroviral naïve until day 1,854 the mean estimated time for start of ART for the CHI group. Lines are simulations. The markers at the first time point (day 135) denote values prior to ART for the PHI group while the second set of markers at day 1,854 denote values prior to ART for the CHI group. Dashed lines in the HIV RNA panels denote infectious components. Coloured lines in the Integrated HIV DNA panel denote infectious components within each cell phenotype. The phenotypes are resting (R), resting and dividing (R^+), activated (A), and activated and dividing (A^+) for each of uninfected cells, or cells containing linear, 2-LTR, or integrated HIV DNA. In the Total HIV DNA panels each of the phenotypes are combined into resting (blue) or activated (red).
