# Supplementary material for: HIV dynamics linked to memory CD4+ T cell homeostasis
Source: PLoS One. 2017 Oct 19;12(10):e0186101. doi: 10.1371/journal.pone.0186101 (PMC5648138; doi:10.1371/journal.pone.0186101)
Supplement: S2 Fig — (PDF) [file pone.0186101.s007.pdf]

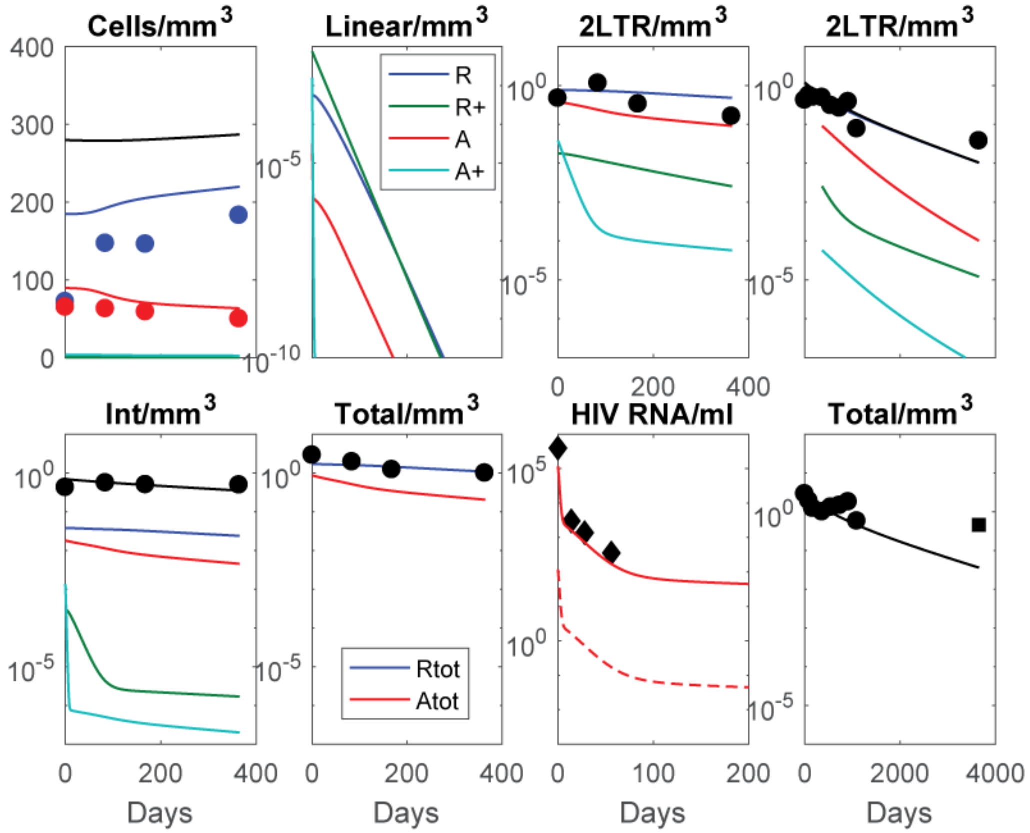

S2 Fig. Simulation of a nonRAL regimen.

Dynamics of cells, HIV DNA and HIV RNA under a nonRAL regimen. The first 3 columns show levels over the first year while the last column shows 2-LTR and Total HIV DNA/mm^3 dynamics over 10 years of nonRAL ART – with the 10 year data points obtained from [1]. The HIV RNA data were obtained from [2], while the cell and HIV DNA data are values for the CHI group (with the RAL regimen). In the HIV RNA panel, the solid line denotes total pVL while the dashed line shows the infectious component.

References

1. Søgaard OS, Graversen ME, Leth S, Olesen R, Brinkmann CR, Nissen SK, et al. The Depsipeptide Romidepsin Reverses HIV-1 Latency *In Vivo*. PLoS Pathog. 2015;11(9):e1005142. doi: 10.1371/journal.ppat.1005142.
2. Murray JM, Emery S, Kelleher AD, Law M, Chen J, Hazuda DJ, et al. Antiretroviral therapy with the integrase inhibitor raltegravir alters decay kinetics of HIV, significantly reducing the second phase. AIDS. 2007;21(17):2315-21. Epub 2007/12/20. doi: 10.1097/QAD.0b013e3282f12377. PubMed PMID: 18090280.
